# A porcine kidney-derived clonal cell line with clear genetic annotation is highly susceptible to African swine fever virus

**DOI:** 10.1186/s13567-024-01300-2

**Published:** 2024-04-04

**Authors:** Hua Cao, Mengjia Zhang, Zheyu Liao, Dongfan Li, Xinglin He, Hailong Ma, Pengfei Li, Xuexiang Yu, Guiqing Peng, Shengsong Xie, Qigai He, Wentao Li

**Affiliations:** 1 National Key Laboratory of Agricultural Microbiology, College of Veterinary Medicine, Huazhong Agricultural University, Wuhan, 430070 China; 2 Key Laboratory of Prevention and Control for African Swine Fever and Other Major Pig Diseases, Key Laboratory of Development of Veterinary Diagnostic Products, Ministry of Agriculture and Rural Affairs, Wuhan, 430070 China; 3 Hubei Hongshan Laboratory, Wuhan, 430070 China; 4 Key Laboratory of Agricultural Animal Genetics, Breeding and Reproduction of Ministry of Education and Key Lab of Swine Genetics and Breeding of Ministry of Agriculture and Rural Affairs, Huazhong Agricultural University, Wuhan, 430070 China

**Keywords:** African swine fever virus (ASFV), LLC-PK1, genome editing

## Abstract

African Swine Fever virus (ASFV), the causative agent of African swine fever, is a highly lethal hemorrhagic virus affecting domestic pigs and wild boars. The primary target cells for ASFV infection are porcine alveolar macrophages (PAMs), which are difficult to obtain and maintain in vitro, and less subjective to genetic editing. To overcome these issues and facilitate ASFV research, we obtained a subclonal cell line PK1-C5 by subcloning LLC-PK1 cells that support stable ASFV proliferation. This consequential cell line exhibited high ASFV infection levels and similar viral growth characteristics to PAMs, while also allowing high-efficiency genomic editing through transfection or lentivirus transduction of Cas9. Taken together, our study provided a valuable tool for research aspects including ASFV-host interactions, pathogenicity, and vaccine development.

## Introduction

African swine fever virus (ASFV) is notorious for affecting domestic pigs and wild boars worldwide, leading to severe and fatal hemorrhagic diseases with nearly 100% mortality [1–3]. ASFV belongs to the genus *Asfivirus* of the *Asfarviridae* family, which is a group of linear, double-stranded DNA virus with a genome of 170–193 kb in size [4]. The first case of ASFV infection in China was reported in August 2018, while the epidemic spread rapidly throughout the nation shortly after, causing enormous economic losses to the swine breeding industry [5]. Thus far, no clinically effective vaccine or antivirals are considered applicable in terms of both safety and effectivity, while the key approaches for controlling viral outbreaks are quarantine and slaughter [6, 7]. This situation demands comprehensive research on ASFV etiology, such as characterization of viral virulence factors and elucidation of detailed viral-host interaction mechanism [8]. One outstanding obstacle is the lack of susceptible cell lines.

ASFV primarily replicates in porcine alveolar macrophages (PAMs) or porcine blood monocyte cells (PBMCs), both of which require time-consuming imminent isolation from pigs and cannot be continuously sustained in tissue culture [6, 9, 10]. Generation of primary PAMs also has a higher chance of inducing microbial contaminations such as Mycoplasma hyopneumoniae (Mhp) and porcine circovirus type 2 (PCV2), which are unsuitable for mass production of vaccines [9]. Current solutions that involve immortalized cell lines include utilization of wild boar lung cell line (WSL), genetically modified porcine kidney (PK15) cells and graduate adaptation of ASFV to human embryo kidney (HEK) cells [11–13]. However, to assist genuine investigation on virus and host factors, it is preferred to use unmodified cell lines originating from the appropriate host together with proper genetic annotation, while viruses shall not rapidly lose fitness upon tissue culture adaptation.

In recent years, immunoprecipitation spectrometry (IP-MS) approach has been utilized and a series of host factors that involved in ASFV immune invasion and replication were identified thereafter [14–16]. These findings provided valuable insights into the mechanisms of ASFV infection and druggable targets. However, as ASFV is a large DNA virus with a complex genome that encodes multiple viral proteins [17], successful infection may partake a synergistic effect upon multiple host factors. CRISPR/Cas9 high-throughput screening have shown potential for resolving the function of genes associated with different phenotypes, and been widely applied to investigate host factors associated with viral adsorption, internalization and replication [18–20]. Successful implementation of the CRISPR/Cas9 screening technique requires a suitable in vitro infection model, whereas virus infection shall induce complete cell death within a short time period [21].

In the present study, we validated a porcine cell line that could effectively accommodate ASFV proliferation. Designated LLC-PK1-Clone 5 (in brief, PK1-C5), this cell line is derived from a LLC-PK1 single cell colony with clear genetic annotation [22], of which different types of ASFV exhibited similar growth properties in this cell line as in PAMs. Meanwhile, lentiviral transduction and CRISPR/Cas9 genome editing experiments indicated that both PK1-C5 and the replicating ASFV within are subject to genetic modification via conventional methods, which will facilitate further characterization of ASFV infection.

## Materials and methods

### Biosafety statement and facility

All experiments involving active ASFV infection were carried out in the biosafety level 3 (BSL-3) facility at Huazhong Agricultural University (Wuhan, China) with the approval by the Ministry of Agriculture and Rural Affairs of the People’s Republic of China. Prior to extraction and detection of viral genomic DNA in BSL-2 laboratory, viruses were first inactivated in a BSL-3 laboratory, and the inactivated samples were transferred afterwards.

### Cells and viruses

HEK-293 T (ATCC No. CRL-3216), LLC-PK1 (ATCC No. CL-101), ST (ATCC No.CRL-1746), PK15 (ATCC No. CCL-33), 3D4/21 (ATCC No. CRL-2843), IPI-2I, IPEC-J2 and NPTR cell lines were retrieved from the cell line database in our lab. All cells were maintained at 37 °C and 5% CO_2_ in Dulbecco’s Modified Eagle Medium (DMEM, Gibco, Waltham, MA, USA) supplemented with 10% (v/v) fetal bovine serum (FBS) (ExCell Bio, Shanghai, China) and 1% (v/v) penicillin streptomycin (pen/strep, Life Technologies). The primary porcine alveolar macrophages (PAMs) were obtained from 20 to 30-day-old piglets, cultured and maintained at 37 °C and 5% CO_2_ in RPMI 1640 medium (Gibco, Waltham, MA, USA) supplemented with 10% FBS (ExCell Bio, Shanghai, China). The ASFV strains used in the present study include genotype I strain LC12, genotype II strain SXH1, and genotype II strain 0428C without RBC adhesion capability. All strains were isolated from clinical samples using PAMs, and propagated for at least three passages.

### Reagents and antibodies

The monoclonal antibodies against ASFV p72, p30, and p54 [23] were prepared in our laboratory for western blotting and immunofluorescence assay (IFA). Antibodies and dyes were purchased, including DAPI (Beyotime, Shanghai, China), Alexa Fluor®488 Donkey anti Mouse IgG (antGene, Wuhan, China), Peroxidase-AffiniPure Goat Anti-Human IgG (H + L) (Jackson ImmunoResearch Inc, West Grove, PA, USA), HRP Goat Anti-Mouse IgG (ABclone, Wuhan, China). Primers and probes for gene amplification were synthesized by Sangon Biotech (Sangon, Shanghai, China).

### Virus infection and titration

To compare the infection efficiency of ASFV in different porcine cell lines, PAMs, LLC-PK1, ST, NPTR, IPI-2I, IPEC-J2, PK15, 3D4/21 and LLC-PK1-clone cells were seeded and infected with ASFV using a MOI of 2. The cells were fixed with 4% paraformaldehyde at 24 h post-infection (hpi), and infection efficiency was determined by the rate of positive signals using immunofluorescence assay (IFA).

To analyze the proliferative stability of ASFV in PK1-C5 cells, PK1-C5 cells in a T25 cell culture flask was infected with ASFV (MOI = 2). After incubation at 37 °C with 5% CO_2_ for 3 days, the mixture of cell and supernatant was collected and stored at −80 °C (designated as P1). The ASFV was serially passaged on PK1-C5 cells in a T25 flask with 1 mL of previously passaged virus for 15 generations under the same condition [11]. Next, PK1-C5 cells and PAMs were infected with virus dilutions of P1, P5, P10, and P15 at MOI = 1. At 48 hpi, virus infectivity was validated using IFA assays.

To compare the growth characteristics of the virus on PAMs and PK1-C5 cells, both cells were inoculated with ASFV SXH1 strain at MOI = 0.5. After incubating at 37 °C with 5% CO_2_ for 2 h, the inoculum was discarded, and the cells were washed twice with PBS. Fresh culture medium was then supplemented to the cells. The mixture of cells and supernatant were collected at 12, 24, 36, 48, 60, 72, 84, and 96 hpi, they were stored at -80 °C for further analysis. The viral titers were determined respectively in PK1-C5 and PAMs and expressed as TCID_50_/mL.

To compare the difference between the progeny virion production in PAMs and PK1-C5 cells, both cells were inoculated with ASFV at MOI = 1 and cell supernatants were collected at 12, 14, 16, 18, 20, 22, 24 and 26 hpi, and viral genome copy numbers were analyzed using qPCR [26].

To determine the titer of the virus, PAMs or PK1-C5 cells were seeded in a 96-well plate and incubated at 37 °C with 5% CO_2_ for 12 h. A tenfold dilution of the virus sample was prepared in the culture medium. The virus dilution was mixed well by pipetting and added to the corresponding wells. The plate was then incubated at 37 °C with 5% CO_2_ for 48 h. Immunofluorescence assay (IFA) was performed using a monoclonal antibody against ASFV p30 protein. The wells showing positive IFA signals were documented, and TCID_50_ (50% tissue culture infective dose) was calculated using the Reed-Muench method [24].

### Lentivirus and cell line preparation

The gRNA targeting ASFV B646L (p72) gene was designed using CRISPR design tool from ZHANG LAB. p72-gRNA-1 (AGATACGTTGCGTCCGTGATAGG) and p72-gRNA-2 (GTGATAAAGCGCTCGCCGAAGGG) were cloned in the pSpCas9n(BB)-2A-Puro plasmid (addgene, plasmid #48141) to generate lentivirus by cotransfect psPAX2 and pMD2G plasmid in HEK-293 T cells. Prepared lentivirus was used to transduce PK1-C5 cells for the generation of a cell line stably expressing the Cas9 protein and gRNAs.

### Immunofluorescence assay

Cells were washed three times with phosphate-buffered saline (PBS), fixed with 4% paraformaldehyde solution at room temperature (RT) for 15 min, permeabilized with 0.1% Triton X-100 in phosphate-buffered saline (PBS) at RT for 15 min, and then blocked with 5% bovine serum albumin (BSA) for 1 h at RT. Subsequently, the cells were incubated with a monoclonal antibody against ASFV-p30 protein at RT for 1 h, followed by three washes with PBS containing 0.5% Tween-20. Next, the cells were incubated with Alexa Fluor 488-conjugated donkey anti-mouse IgG (antGene, Wuhan, China) for 1 h. The cell nuclei were stained with 4′,6-diamidino-2-phenylindole (DAPI) (Beyotime, Shanghai, China). Fluorescence images were captured using an inverted fluorescence microscope (SOPTOP, Shanghai, China).

### Western blotting

Cells were harvested with lysis buffer (Beyotime, Shanghai, China) supplemented with 1 mM PMSF on ice. The lysates were heated at 95 °C for 10 min with 5 × protein loading buffer to denature the samples. Proteins were separated using 12% SDS-PAGE and transferred on polyvinylidene difluoride (PVDF) membranes (Bio-Rad, 0.45-μm pore size). The membranes were blocked for 2 h at RT with 5% skimmed milk, and then incubated with primary antibody for 1 h at RT, followed by washing four times with TBST (each wash for 5 min). The membranes were incubated with horseradish peroxidase (HRP)-conjugated goat anti-mouse IgG or anti-human IgG secondary antibody at RT for 45 min. After four washes, the membranes were visualized using a chemiluminescent substrate (Bio-Rad, CA, USA).

### Electron microscopy

To examine the virion morphology, PAMs and PK1-C5 cells were seeded in 6-well plates and infected with ASFV at MOI = 1. At 36 hpi after infection, virus-infected cells were collected and observed via electron microscopy with reference to previously described method [25].

### Real-time qPCR analysis

ASFV genomic DNA was extracted from cell culture supernatant and cells using the TIANamp Genomic DNA Kit (Tiangen Biotech, Beijing, China). The relative quantity of viral DNA was determined using the Animal Detection U + Probe qPCR Super PreMix (Vazyme, Nanjing, China) with primers and probes targeted the p72 gene of ASFV. Total RNAs were extracted using TRIzol reagent (Invitrogen, USA), and 500 μg of total RNA of per sample was reverse transcribed into cDNA using the HiScript II 1^st^ Strand cDNA Synthesis Kit-R211 (Vazyme, Nanjing, China). AceQ Universal SYBR qPCR Master Mix-Q511(Vazyme, Nanjing, China) were used in the qPCR assay. The glyceraldehyde-3-phosphate dehydrogenase (GAPDH) gene was used as an endogenous control and the relative expression of mRNA was calculated based on the comparative cycle threshold (C_T_) (2^−ΔΔCT^) method Real-time RT-PCR was performed with the CFX Connect real-time PCR detection system (Bio-Rad, CA, USA). Gene-specific primer and probe sequences are listed in Table 1.

**Table 1:** **Primers used in this study**

Primer sets	Sequences (5’–3’)
B646L	F: CTGCTCATGGTATCAATCTTATCGAR: GATACCACAAGATC(AG)GCCGT
B646L-Probe	FAM-CCACGGGAGGAATACCAACCCAGTG-3′-BHQ1
IFN-β	F: CAACAAAGGAGCAGCAAT R: TGGAGCATCTCGTGGATA
ISG15	F: GGTGCAAAGCTTCAGAGACC R: GTCAGCCAGACCTCATAGGC
ISG56	F: CCCACTTCTGTCTTACTGC R: TACATTCTTGCCAGGTCTA
GAPDH	F: CCTTCCGTGTCCCTACTGCCAAC R: GACGCCTGCTTCACCACCTTCT

### Flow cytometry

LLC-PK1 cells were subjected to trypsin digestion, followed by filtration through a 100 μm cell strainer (Biosharp, Anhui, China). Subsequently, the cells were washed once with DMEM culture medium containing 1% pen/strep (Gibco, Waltham, MA, USA). Single-cell sorting mode of the MoFlo XDP (Beckman Coulter, Miami, FL, USA) flow cytometer was utilized to sort the cells into a 96-well cell plate.

### Cell viability assay

PK1-C5 cells were cultured in a 96-well plate with a cell density of 2.5 × 10^4^ cells/well and then infected with ASFV (MOI = 3). The supernatant from the infected cells was collected at 24, 48 and 72 hpi. Lactate dehydrogenase (LDH) release of the samples was measured using the LDH Cytotoxicity Assay Kit (Beyotime, Shanghai, China) according to the manufacturer's instructions.

### Statistical analysis

Data are shown as the mean ± standard deviation (SD). Statistical significance was determined using GraphPad Prism 9 software, and *P*-values were calculated using Student’s *t*-test and one-way ANOVA. Asterisks in the figures indicate the levels of significance (*, *P* < 0.05; **, *P* < 0.01; ***, *P* < 0.001).

## Results

### Biological properties of ASFV in swine-derived cells

**Figure 1:**
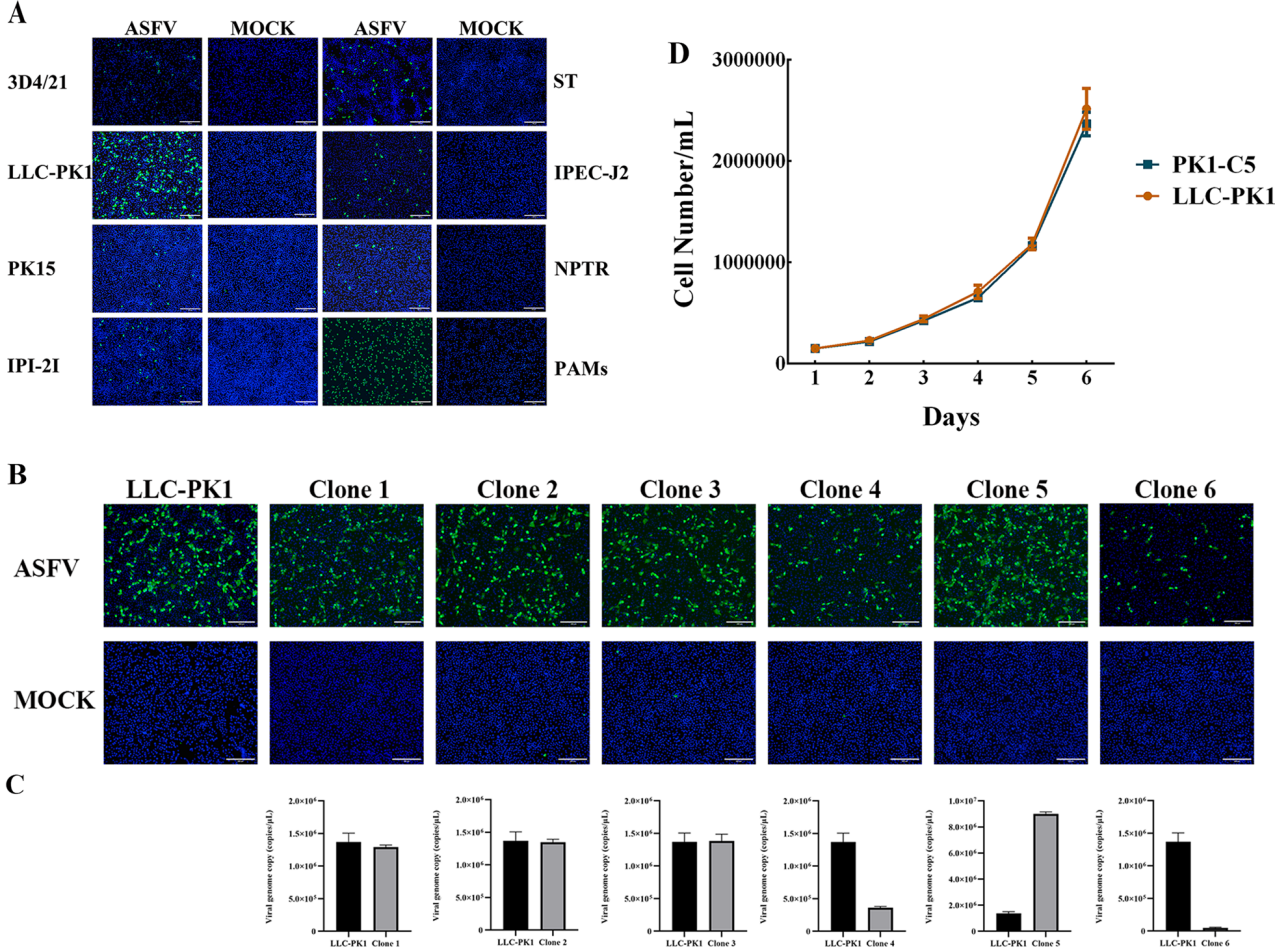
**A cloned LLC-PK1 cell line showed increased ASFV susceptibility. A** ASFV infection in different swine-derived cell lines. **B** ASFV infectivity in parental LLC-PK1 or its subclones were examined by IFA with a monoclonal antibody against the ASFV p30 protein. Scale bar = 200 μm. **C** ASFV infectivity in parental LLC-PK1 or its subclones were examined by qPCR. **D** The number of parental LLC-PK1 cells and Clone 5 (PK1-C5) monoclonal cells at different time points. Scale bar = 200 μm.

Different swine-derived cells are inherently susceptible to ASFV at different extents. In our present experimental setting, we first evaluated the infection efficiency of ASFV in different porcine cell lines, including LLC-PK1 (from porcine kidney), ST (from porcine testis), NPTR (from porcine trachea), IPI-2I (from porcine intestine), IPEC-J2 (from porcine small intestine), PK15 (from porcine kidney), 3D4/21 (from porcine macrophage) and freshly obtained PAMs. After 24 h of infection with ASFV strain SXH1 (MOI = 2), cells were fixed, and infectivity was examined via immunofluorescence (IFA) assay using a monoclonal antibody against the ASFV p30 protein [23]. The results indicated that the expression of ASFV p30 protein could be detected in all the tested porcine cell lines, while the infection level of ASFV in LLC-PK1 cells was lower than that in PAMs, but also significantly higher than that in other porcine cell lines (Figure 1A).

The infection result indicates innate heterogeneity of LLC-PK1 cells upon ASFV infection. To obtain uniformed, high infectivity LLC-PK1 clones, this cell line was subcloned for two rounds by flow cytometry to select for monoclonal cells exhibiting higher ASFV susceptibility. As shown in Figures 1B and C, after ASFV-SXH1 (MOI = 2) infection, different LLC-PK1 clones displayed diverse susceptibilities, whereas the infection efficiency in the clone 5 (PK1-C5) monoclonal cell line was the highest. We next analyzed the growth characteristics of PK1-C5 cells by measuring the number of uncloned LLC-PK1 cells and PK1-C5 cells at different time points (Figure 1D). The results showed that the cell growth rate and number of PK1-C5 cells were consistent with those of uncloned LLC-PK1 cells. Therefore, the PK1-C5 cell clone showed genuine properties and was selected for follow-up study.

### Efficient replication of ASFV in PK1-C5

**Figure 2:**
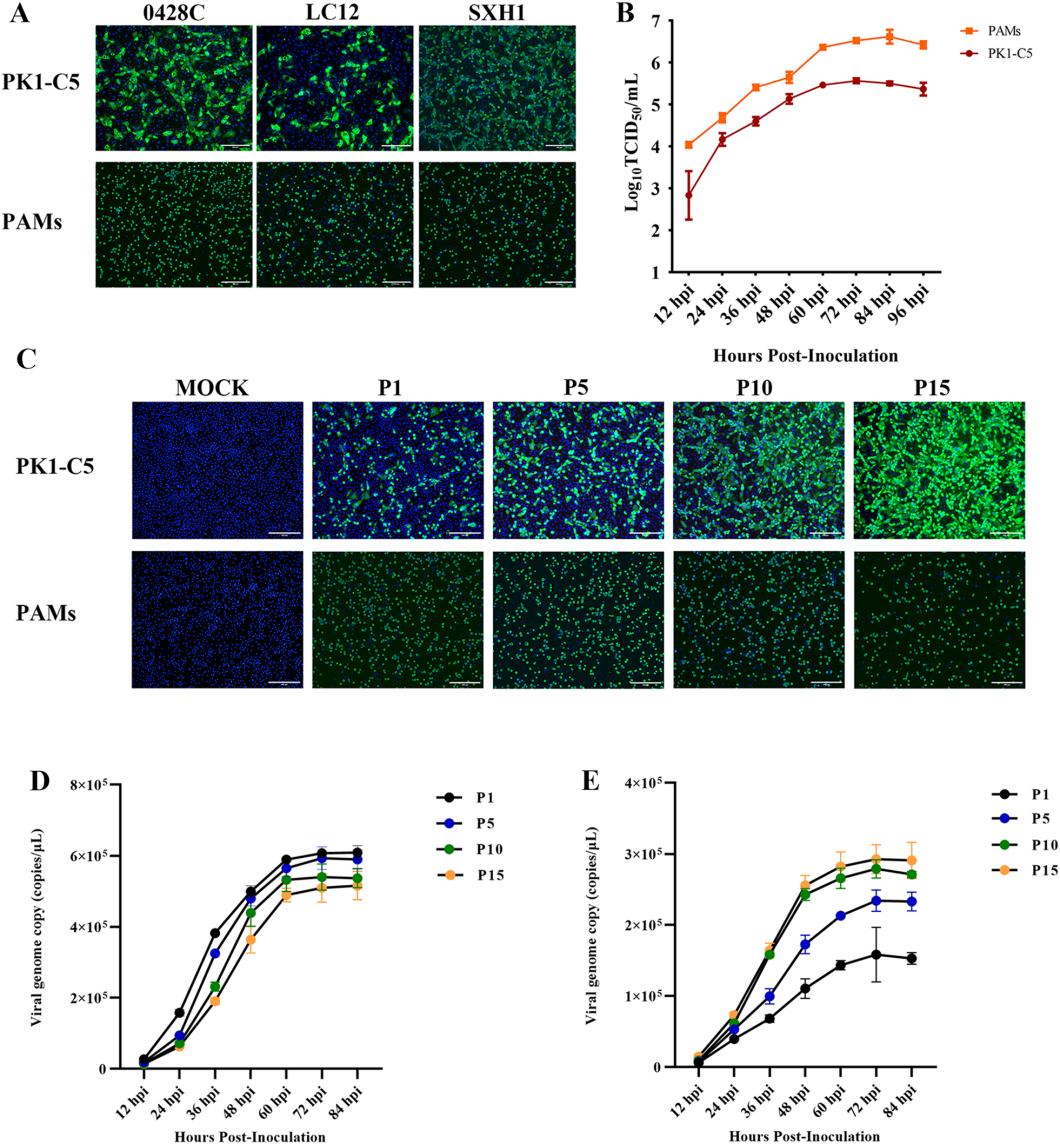
**Infection and replication of ASFV in PK1-C5. A** Visualization of infection with ASFV field strains in PK1-C5 cells and PAMs by immunostaining with an antibody against viral p30 protein (green). Scale bar = 200 μm **B** PK1-C5 cells and PAMs were infected with P1, P5, P10 and P15 viruses at MOI = 1, while IFA was utilized to detect ASFV proliferation in PK1-C5 cells through consecutive virus passages. Scale bar = 200 μm. **C** Growth curve of ASFV in PK1-C5 cells and PAMs. **D** In vitro growth kinetics of passaged ASFV (P1, P5, P10, P15) in PAMs. **E** In vitro growth kinetics of passaged ASFV (P1, P5, P10, P15) in PK1-C5 cells.

To determine the levels of ASFV infection with ASFV field strains, we infected PAMs and PK1-C5 cells with ASFV isolate LC12, SXH1 and 0428C at MOI of 1. Infection was terminated at 48 hpi, and IFA was performed using an ASFV p30-specific monoclonal antibody (Figure 2A). The results showed that these isolates were able to infect PK1-C5 cells at high levels, while significant fluorescence signal was observed in each infection setting.

To systematically assess the replication of ASFV on PK1-C5, we infected PAMs and PK1-C5 cells side by side with ASFV-SXH1 (MOI = 0.5), and the mixture of cells and supernatant were collected at 12-h intervals up to 96 hpi. The viral titers were titrated and the corresponding virus growth curve was plotted (Figure 2B). The results showed that the replication trend of ASFV in the two cells was similar, while virus titers peaked 72 h after infection with PK1-C5 cells (1 × 10^5.56^ TCID_50_/mL, 0.1-fold to that of PAMs) and then started to decrease. Such findings indicate that ASFV can replicate effectively on PK1-C5 cells.

In addition, we analyzed the stability of ASFV proliferation in PK1-C5 cells through consecutive virus passages. ASFV was serially passed on PK1-C5 cells for 15 consecutive times under the same condition, while PK1-C5 cells and PAMs were infected afterwards with P1, P5, P10 and P15 viruses at MOI = 1 to study the viral progeny. IFA results at 48 hpi showed a gradual increase in the adaptation of ASFV to PK1-C5 cells (Figure 2C). Meanwhile, we have analyzed the growth kinetics of P1, P5, P10 and P15 virus on both PK1-C5 cells and PAMs based on the relative genomic copies. As shown in Figures 2D and E, the growth kinetics of passaged ASFV in PAMs and PK1-C5 cells were similar, reaching a plateau at 72 hpi.

### Analysis of virion stability

**Figure 3:**
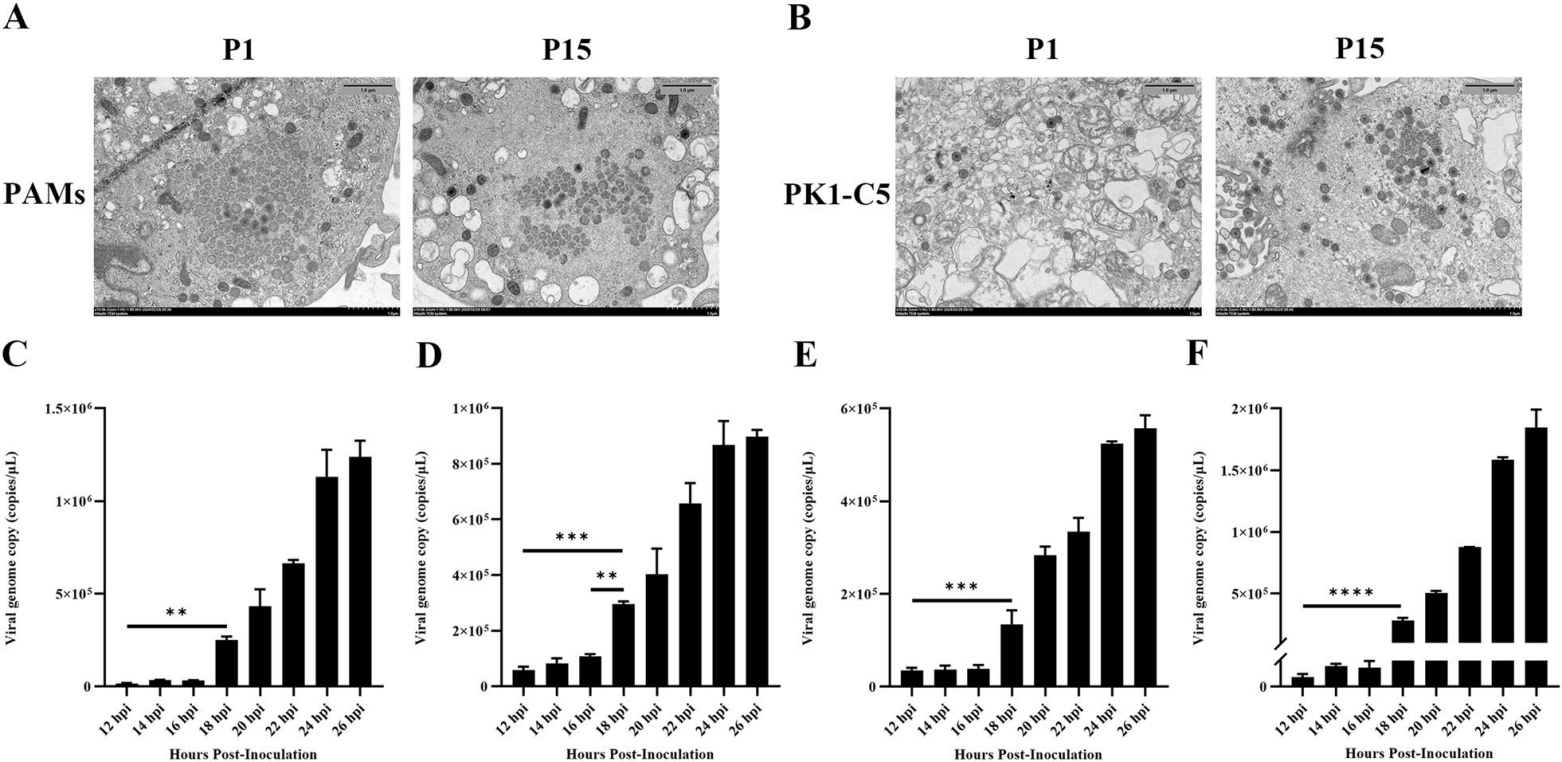
**Virion morphology and the time of one life cycle of ASFV infection in PAMs and PK1-C5. A** Virion morphology of P1 and P15 virus were observed by electron microscopy in PAMs. Scale bar = 1.0 μm. **B** Virion morphology of P1 and P15 virus were observed by electron microscopy in PK1-C5 cells. Scale bar = 1.0 μm. **C**, **D** The growth kinetics of P1 and P15 virus on PAMs. **E**, **F** The growth kinetics of P1 and P15 virus on PK1-C5 cells.

To validate ASFV stability after continuous passage on PK1-C5, we analyzed the morphology of the virion via electron microscopy, and the results showed that the morphology of the ASFV P15 is similar with the parental virus on both cell types (Figures 3A and B). In addition, to compare the growth kinetics of ASFV-P1 and -P15, we collected cell supernatants of infected cells at 12 hpi, 14 hpi, 16 hpi, 18 hpi, 20 hpi, 22 hpi and 24 hpi, and examined viral genome copies by qPCR. As shown in Figures 3C–F, the viral growth in the supernatant is low before 18 hpi, and then increased significantly from 18 hpi. This finding is consistent with the previous report that the cycle of ASFV replication is around 18 h [26, 27], which is seemingly also shared by P1 and P15 viruses on both cell types. Besides, the complete genomic sequences of the ASFV-P15 were obtained and compared to the genome of the parental ASFV. ASFV-P15 accumulated five nucleotide mutations at passage 15 in PK1-C5 cells relative to parental ASFV. A C-to-T mutation in the G1211R gene results in a A706V substitution in the protein. Other point mutations are C-T mutations in the B407L gene results in a D124N and V126I substitution in the in the protein. A base insertion occurred at 47nt of DP60R gene, which affected the protein translation.

### High efficiency genome editing in PK1-C5

**Figure 4:**
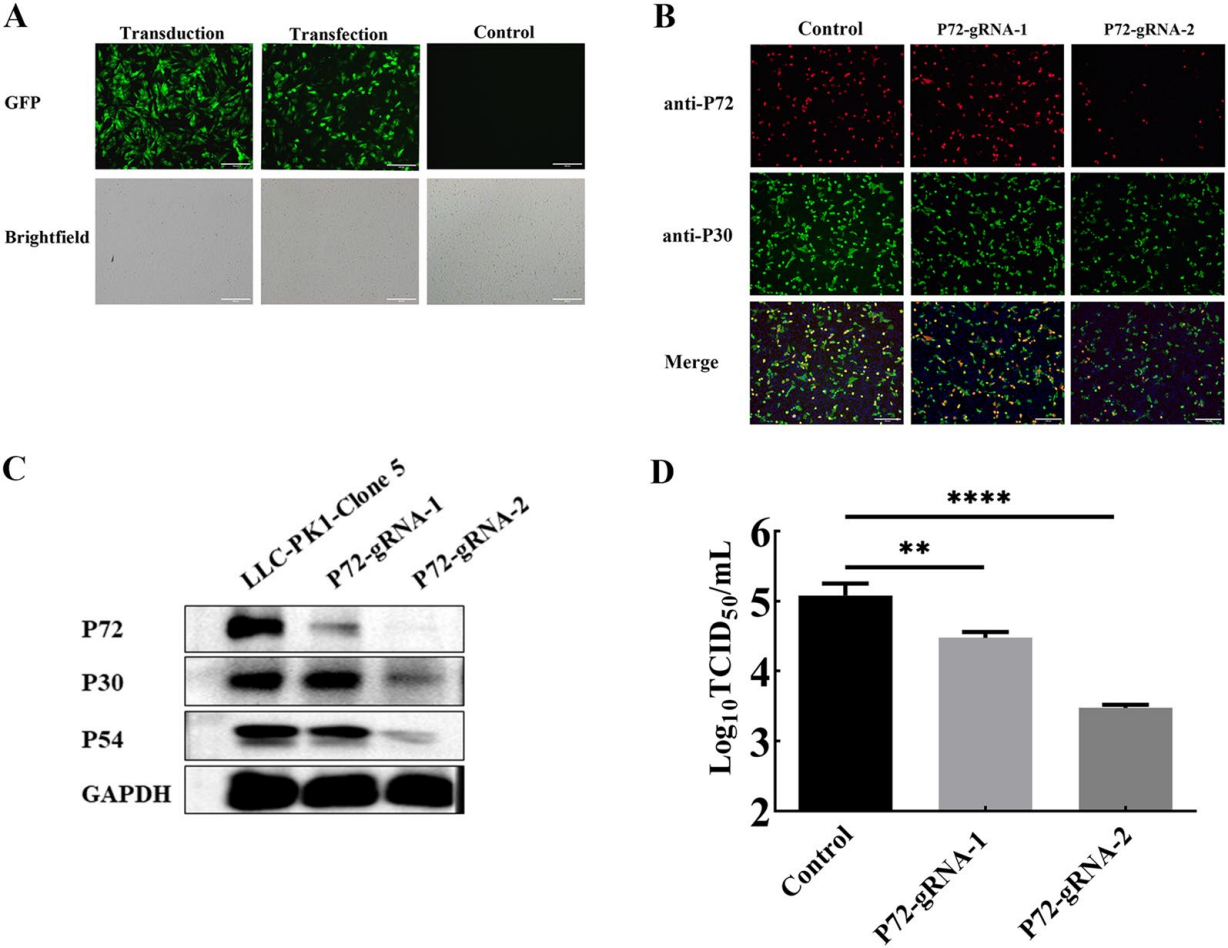
**Efficiency of gene editing in PK1-C5. A** Transfection and lentiviral transduction efficiency in PK1-C5 cells. Scale bar = 200 μm. **B** Immunostaining for ASFV p30 and p72 protein in ASFV infected PK1-C5 cells and p72 gene targeting sgRNA transduced (PK1-C5-P72^KO^) cells. Scale bar = 200 μm. **C** Western blot for detection of ASFV p30, p54 and p72 proteins in ASFV infected PK1-C5 cells and potential PK1-C5-P72^KO^ cells. **D** Virus titers in PK1-C5 cells and PK1-C5-P72^KO^ cells at 36 hpi.

To evaluate the practicability of gene editing in PK1-C5, a eukaryotic expression plasmid carrying green fluorescent protein (GFP) was first used to transfect PK1-C5 cells, and green fluorescence was observed in more than 30% of the cells 24 h after transfection (Figure 4A). In addition, lentiviral transduction of PK1-C5 cells was conducted, and positive signals were observed in more than 50% of the cells 48 h post-transduction (Figure 4A). Additionally, two guide RNAs (gRNAs) targeting the ASFV B646L gene, p72-gRNA-1 and p72-gRNA-2, were further designed and packaged in a lentiviral transduction setting for CRISPR/Cas9 genome editing. The PK1-C5 cells was then transduced, and a pre-infection PK1-C5-P72^KO^ cell line was obtained through resistance screening with puromycin. After transduction and selection, the remaining cells were enriched and infected with ASFV-SXH1 (MOI = 1). IFA and Western blot analysis was performed at 24 hpi or 48 hpi. The results showed that p72-gRNA-2 could effectively induce the editing of the ASFV B646L gene, resulting in limited expression of the p72 protein, henceforth reducing the production of viral progeny by approximately 100-fold (Figures 4B–D).

### Imminent cell death of PK1-C5 post ASFV infection

**Figure 5:**
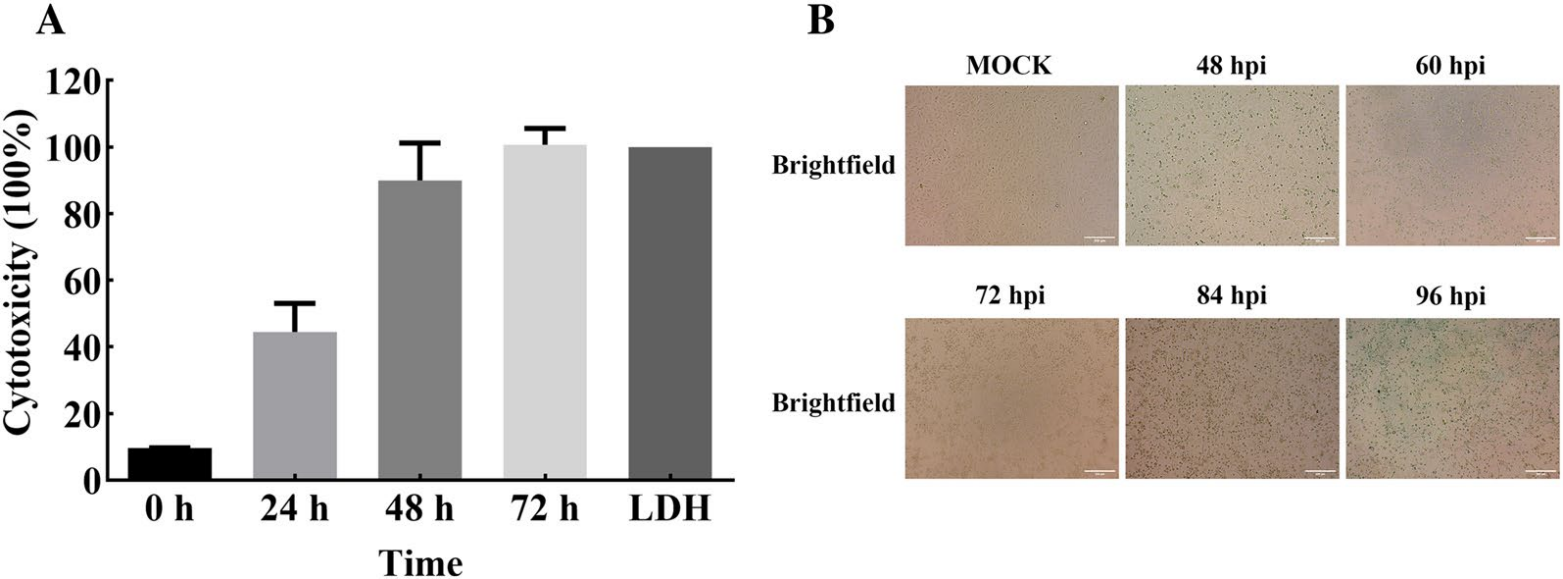
**The cytopathic effect of PK1-C5 cells infected with ASFV. A** LDH release of PK1-C5 cells during ASFV infection at different time point. Data are represented as means ± SDs (*n* = 3). **B** CPE in PK1-C5 cells at 48 h, 60 h, 72 h, 84 h, 96 h after ASFV infection. Scale bar = 200 μm.

PK1-C5 cells were infected with ASFV (MOI = 3), and a cell lactate dehydrogenase (LDH) release assay was utilized to investigate the ASFV-induced damage to PK1-C5 cells. As indicated in Figure 5A, cells released LDH post ASFV infection, while infected cells showed a comparative LDH release to control at 72 hpi. Additionally, full cytopathic effect (CPE) was observed after viral infection, with over 95% of cells showing morphological changes and detachment at 72 hpi (Figure 5B).

### ASFV replication inhibits type I IFN in PAMs and PK1-C5 cells

**Figure 6:**
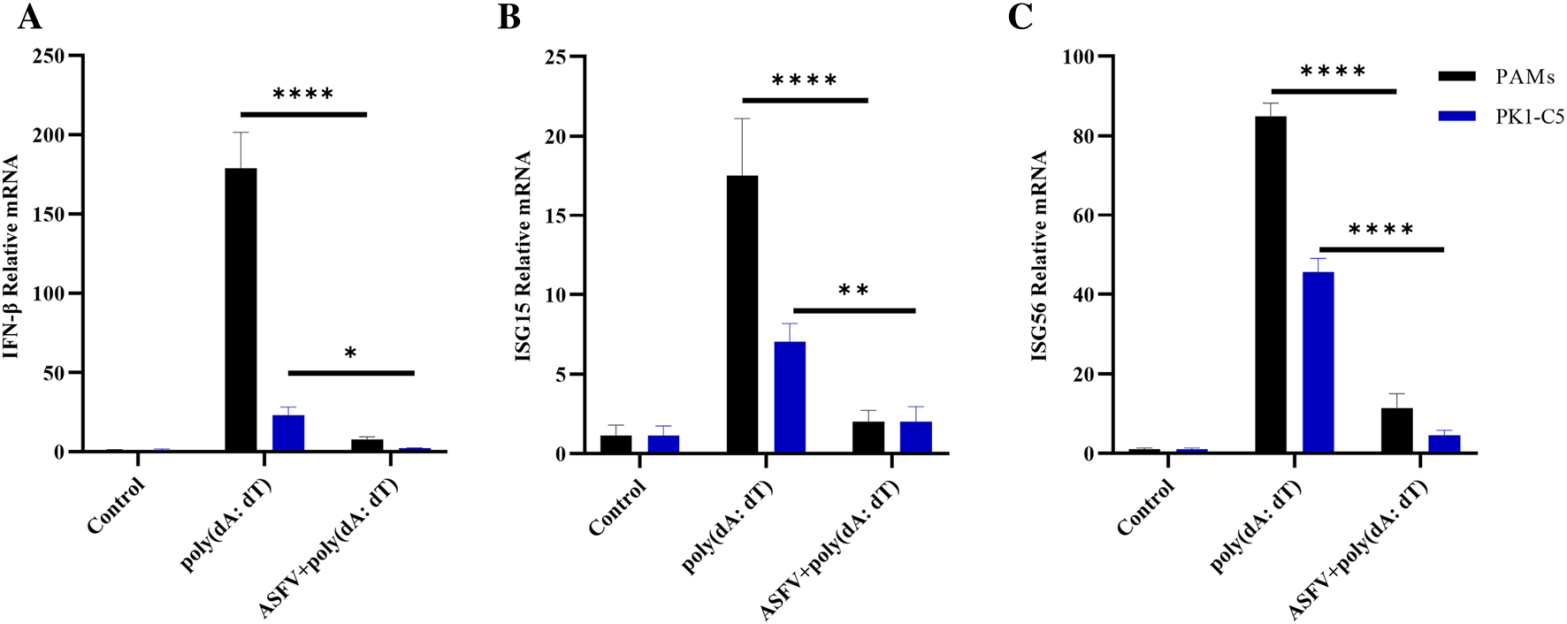
**ASFV replication suppressed IFN-β, ISG15, and ISG56 expression.** PAMs and PK1-C5 cells were mock treated or transfected with poly(dA:dT) for 12 h. The poly(dA:dT)-transfected cells were subsequently mock infected or infected by ASFV (MOI = 1) for 24 h. All the cells were then collected, and levels of IFN-β (**A**), ISG15 (**B**), and ISG56 (**C**) genes were detected using qPCR.

To evaluate the influence of ASFV infection on expression of type I IFN, PAMs and PK1-C5 cells were mock treated, transfected with poly(dA:dT), or transfected with poly(dA:dT) followed by ASFV infection for 24 h. The results indicated that ASFV infection significantly decreased poly(dA:dT)-induced IFN-β, ISG15, and ISG56 mRNA expression (Figures 6A–C). This suggested that ASFV infection inhibited the antiviral effect of the host IFN pathway.

## Discussion

Numerous efforts were implemented on building in vitro infection models that could successfully sustain ASFV for its etiology research and vaccine development. Here, we started by examination of the susceptibility of different swine-derived cells to ASFV infection and found that LLC-PK1 cells exhibited decent infection efficiency with inherent heterogenicity. Subsequently, we performed single cell cloning and showed that the clonal cell line PK1-C5 could support highly stable and efficient ASFV proliferation, henceforth served as a valuable tool for fundamental and applied ASFV research.

Porcine mononuclear macrophages (PAMs) are the previous main target cells of ASFV infection, but they cannot be replicated in vitro, and the production process is complex and costly [28]. In addition, PAM cells obtained from different pigs diverge greatly as each pig has verified genetic background, and usage of those cells for vaccine production is highly inherently limited. Therefore, it is imperative to find a cell line that can support the stable proliferation of African swine fever virus for fundamental research and vaccine development of ASFV.

At present, different cell lines have been used in the study of ASFV, and Vero cells have been widely used in the study of ASFV gene function, viral proteomic analysis, viral transcription and replication [29, 30]. However, as ASFV replicates in Vero cells with increasing titers, they gradually lost its ability to replicate in PAMs in vitro [31]. Similarly, through continuous passage of ASFV in HEK-293 T cells, they found that the left variable region (MGF300, MGF360) of the ASFV genome is progressively missing and the virus has reduced PAMs infectivity [12]. In the present study, we confirmed that the monoclonal selected cell line PK1-C5 is susceptible to ASFV, and showed that a gradual increase in adaptation of the ASFV to grow in PK1-C5 cells (Figures 2C and E). A comparative analysis of the genome of ASFV-P15 and the parent strain revealed that ASFV-P15 accumulated five nucleotide mutations at passage 15 in PK1-C5 cells. The correlation between these mutations and viral adaption to PK1-C5 cells will be addressed in our further studies.

Animal experiments on ASFV indicated that kidneys of infected pigs were hyperemic and swollen, and the presence of virion could be detected in the kidneys [32], which further verified that PK1-C5 could support ASFV infection and proliferation. Interestingly, different LLC-PK1 clones displayed diverse ASFV susceptibilities. Observations as such shall promote differential omics analysis upon LLC-PK1 subgroups, where their biological discrepancies in correspondence with ASFV infectivity shall provide insights key to virus-host interaction.

To date, gene editing of PAMs remains cumbersome due to discontinuous cell passage and genetic heterogenicity [33]. For the moment, studies of the effect of host factors in ASFV infection is mostly achieved by using siRNA knockdown in PAMs, and gene knockout or overexpression in MA104 and other cell lines [14, 15]. Therefore, we evaluated the possibility of gene editing in PK1-C5 cells. Through transfection GFP encoding plasmid and lentiviral transduction, we found that this clonal cell line has high transfection and transduction efficiency (Figure 4A), and stable expression of p72-gRNA and Cas9 proteins in the cell line could significantly reduce virus growth (Figures 4B–D). Apparently, both PK1-C5 and the replicating ASFV within are highly efficient regarding genetic modification, which shall further assist in studies of ASFV virulence and host factors. These findings have significant implications for the rapid construction of genetically modified viruses, manipulation of cell lines to regulate gene expression, and identification of ASFV host factors crucial for efficient infection. CRISPR/ Cas9 based high-throughput screening is widely used to identify key host factors for different viral infections [19, 20]. Implementation of this approach preferably requires cell line that has clear genetic annotation, and untargeted cells to undergo rapid death post virus infection. Upon ASFV infection (MOI = 3), 95% cytopathic death of PK1-C5 cells could be observed at 72 hpi (Figure 5A). Such observations suggest that PK1-C5 cells can be completely killed upon ASFV-SXH1 infection, and this cell line can be utilized as a versatile tool for CRISPR/Cas9 technology to screen host factors necessary for ASFV infection.

In conclusion, we identified and characterized a porcine-derived cell line PK1-C5 that sustains high efficiency and stability of ASFV infection, and can also be genetically edited by conventional laboratory techniques. Utilization of this cell line will contribute to a wider exploration of the pathogenic mechanism of ASFV and open avenues for the development of novel antiviral interventions.

## Data Availability

The datasets analysed in the current study are available from the corresponding author on reasonable request.
